# Supplementary material for: Cytokinin fluoroprobe reveals multiple sites of cytokinin perception at plasma membrane and endoplasmic reticulum
Source: Nat Commun. 2020 Aug 27;11:4285. doi: 10.1038/s41467-020-17949-0 (PMC7452891; doi:10.1038/s41467-020-17949-0)
Supplement: Supplementary file 1 — Supplementary Information [file 41467_2020_17949_MOESM1_ESM.pdf]

Supplementary information for manuscript

Cytokinin fluoroprobe reveals multiple sites of cytokinin perception at plasma membrane and endoplasmic reticulum

Karolina Kubiasová[1,#], Juan Carlos Montesinos[2,#], Olga Šamajová[3], Jaroslav Nisler[4,5], Václav Mik[4], Hana Semerádová[2], Lucie Plíhalová[4,5], Ondřej Novák[5], Peter Marhavý[2,7], Nicola Cavallari[2], David Zalabák[1], Karel Berka[6], Karel Doležal[4,5], Petr Galuszka†, Jozef Šamaj[3], Miroslav Strnad[5], Eva Benková[2,*], Ondřej Plíhal[1,4,5*], and Lukáš Spíchal[4,*]

[1]*Department of Molecular Biology, Centre of the Region Haná for Biotechnological and Agricultural Research, Faculty of Science, Palacký University, Šlechtitelů 27, 783 71 Olomouc, Czech Republic;*

[2]*Institute of Science and Technology (IST), 3400 Klosterneuburg, Austria;*

[3]*Department of Cell Biology, Centre of the Region Haná for Biotechnological and Agricultural Research, Faculty of Science, Palacký University, Šlechtitelů 27, 783 71 Olomouc, Czech Republic;*

[4]*Department of Chemical Biology and Genetics, Centre of the Region Haná for Biotechnological and Agricultural Research, Faculty of Science, Palacký University, Šlechtitelů 27, 783 71 Olomouc, Czech Republic;*

[5]*Laboratory of Growth Regulators, Institute of Experimental Botany of the Czech Academy of Sciences and Faculty of Science of Palacký University, Šlechtitelů 27, 783 71 Olomouc, Czech Republic;*

[6]*Department of Physical Chemistry, Regional Centre of Advanced Technologies and Materials, Faculty of Science, Palacký University, 17. listopadu 1192/12, 771 46 Olomouc, Czech Republic;*

[7]*Umeå Plant Science Centre, Department of Forest Genetics and Plant Physiology, Swedish University of Agricultural Sciences, 90183 Umeå, Sweden.*

†deceased

[#]*These authors contributed equally*

[*]*For correspondence: eva.benkova@ist.ac.at; ondrej.plihal@upol.cz; lukas.spichal@upol.cz*

List of supplementary material

Supplementary Figures 1-7

Supplementary Table 1

Supplementary Methods

Supplementary References

Supplementary Fig 1

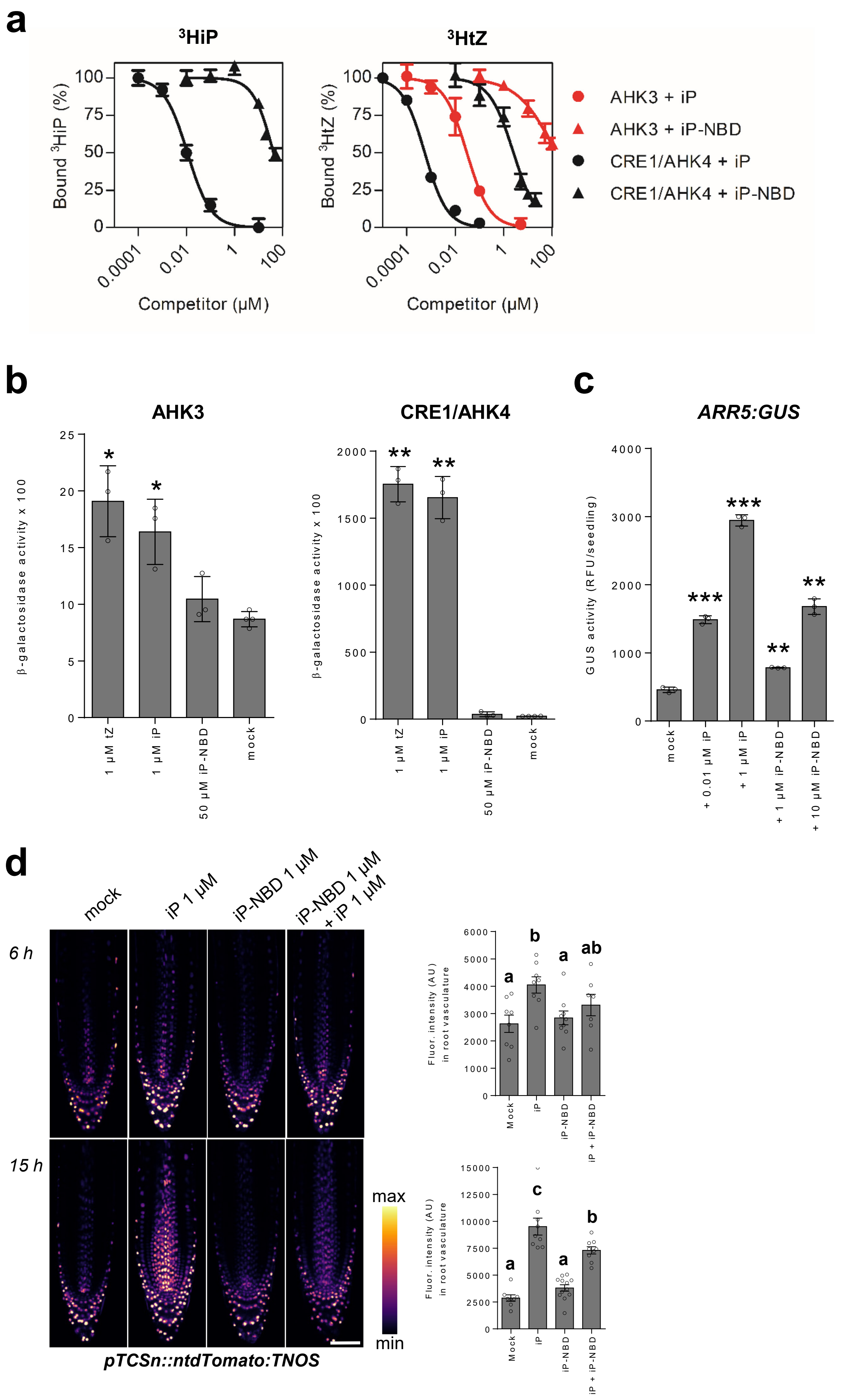

Supplementary Figure 1. Biological characterization of iP-NBD.

a. Competitive binding assay with *Escherichia coli* expressing AHK3 and CRE1/AHK4. Binding of 6 nM [2-^{3}H]iP and 3 nM [2-^{3}H]*t*Z was assayed together with increasing concentrations of of iP-NBD (triangles) and unlabelled iP (circles). The functional inhibition curves for AHK3 and CRE1/AHK4 are presented in red and black, respectively. **b.** Comparison of cytokinin response in *E. coli* (*ΔrcsC, cps::lacZ*) receptor activation assay with recombinant AHK3 and CRE1/AHK4 receptors, triggered by 1 µM *t*Z and 1 µM iP (positive controls) and 50 µM iP-NBD. Mock treatment represents solvent control DMSO (0.1%). The bars represent mean ± s.d., ** = $p < 0.01$, * = $p < 0.05$; by Student's t-test, n = 3. **c.** Quantitative evaluation of β-glucuronidase activity in Col-0 seedlings harbouring *ARR5::GUS* after incubation with 0.01 µM and 1 µM cytokinin N^6-isopentenyladenine (iP), and 1 µM and 10 µM fluoroprobe iP-NBD. Mock treatment represents solvent control DMSO (0.1%). The bars represent mean ± s.d., *** = $p < 0.001$, ** = $p < 0.01$; n = 3 (Student's t-test). **d**. Expression of the cytokinin sensitive reporter *pTCSn::ntdTomato:TNOS* in Arabidopsis roots treated with 1 µM N^6-isopentenyladenin (iP; positive control) and 1 µM iP-NBD for 6 and 15 h. Fluorescence intensity measured in the root vasculature (mean ± s.d., $p < 0.05$ by two-way ANOVA, n > 8 root tip per condition). Scale bar = 50 µm.

Supplementary Fig 2

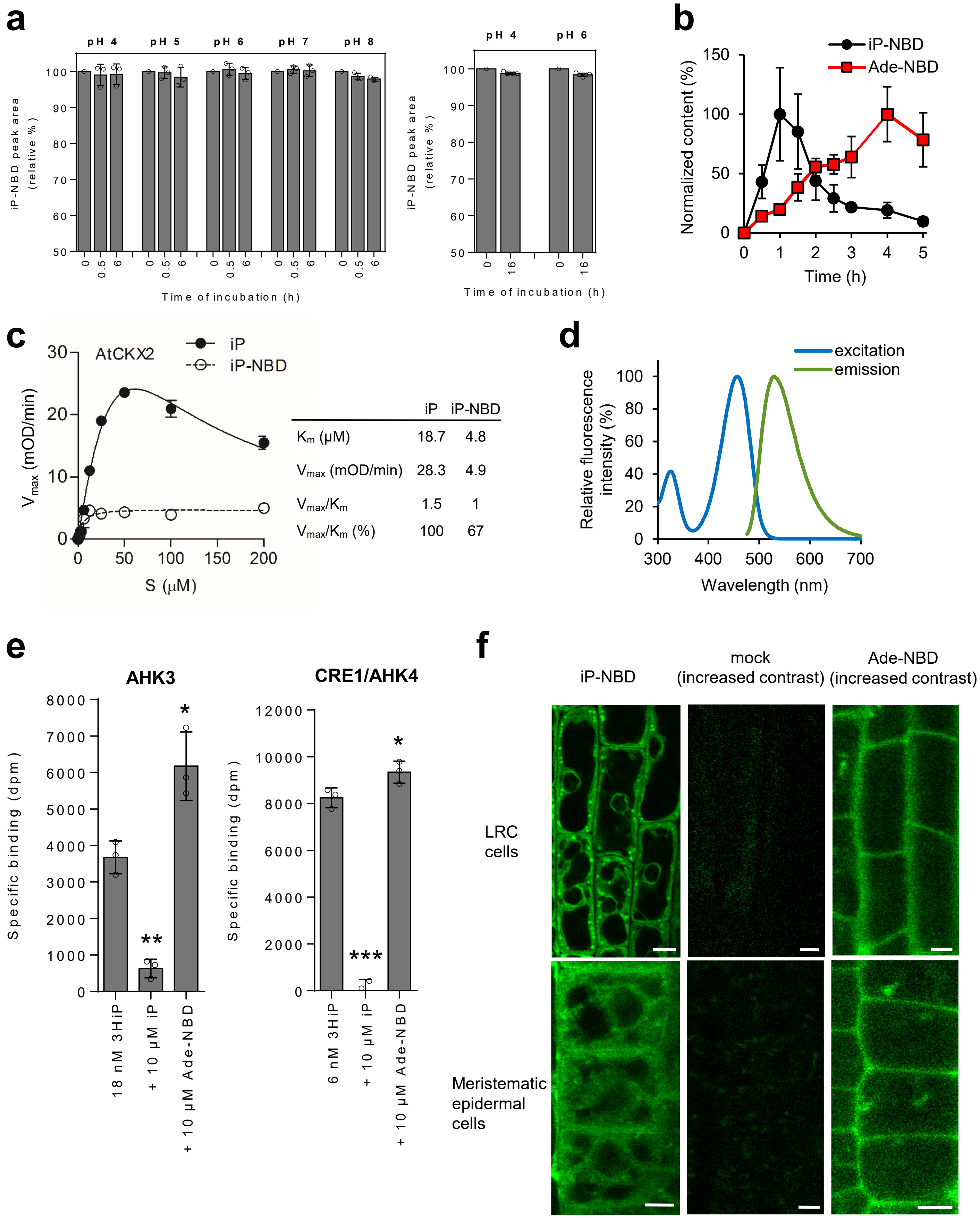

Supplementary Figure 2. Evaluation of iP-NBD biological stability and fluorescence characteristics.

a. *In vitro* pH stability of iP-NBD. Stability of iP-NBD was followed by LC-MS/MS analysis in water solution after 0.5 h and 6 h of incubation in the McIlvaine buffer (pH range 4-8; left panel) and after 16 hours of incubation in the McIlvaine buffer (pH 4 and pH 6; right panel). Bars represent relative peak areas of iP-NBD, which was incubated for given time periods in the respective buffer solution as compared to the iP-NBD control (0 h) at the same concentration (mean ± s.d., n≥3). **b.** *In vivo* iP-NBD stability. iP-NBD was applied to *Arabidopsis* (L*er*) cells suspension and in the timeframe of 0.5-5 h its intracellular processing was followed by quantitative LC-MS/MS analysis using iP-NBD and Ade-NBD (the expected product of side-chain cleavage by endogenous CKXs) as molecular standards (as described in the methods). The values presented in the graph are normalized to respective highest content of both compounds analysed. The highest contents of intracellular iP-NBD and Ade-NBD were 7920 ± 3150 pmol/g and 19633 ± 4903 pmol/g, respectively (FW, mean ± s.d., n = 4). **c.** Kinetics and kinetic parameters of *in vitro* AtCKX2 enzymatic activity estimated using iP and iP-NBD as substrates, respectively, in the concentration range of 1.6 - 200 µM. **d.** Fluoroprobe absorption-emission spectral diagram measured with 100 µM iP-NBD dissolved in 100% ethanol. Absorption (excitation) and emission spectra are reaching their maximal fluorescence intensities in 456 nm (ex) and 528 nm (em). **e.** Competitive binding assay with *Escherichia coli* expressing AHK3 and CRE1/AHK4 with Ade-NBD. Binding of [2-^{3}H]iP (18 nM and 6 nM in the case of AHK3 and CRE1/AHK4, respectively) was assayed together with high excess concentration of Ade-NBD (10 µM), and unlabelled iP (10 µM) as a positive control (mean ± s.d., *** = $p < 0.001$, ** = $p < 0.01$, * = $p < 0.05$; by Student's t-test, n = 3). **f.** Differential localization of iP-NBD and Ade-NBD in Arabidopsis LRC and epidermal cells. Roots were treated for 10 min with iP-NBD or Ade-NBD (5 µM). Roots without any treatment (mock) were used as a control. Scale bar = 5 µm.

Supplementary Fig 3

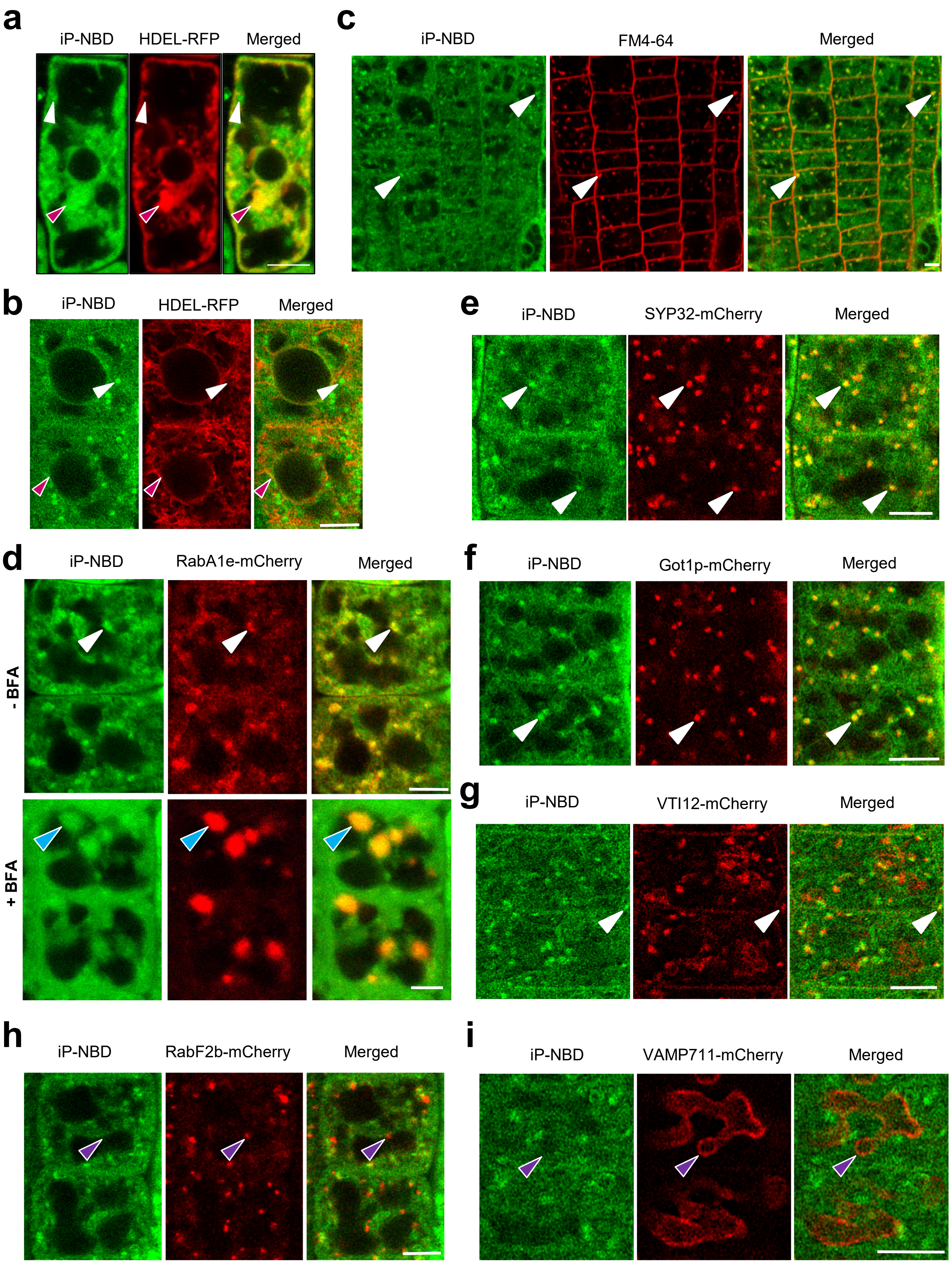

Supplementary Figure 3. Monitoring of iP-NBD subcellular localization in Arabidopsis root cells.

a, b. Monitoring of iP-NBD (green) and ER-marker HDEL-RFP (red) in LRC cells (**a**) and epidermal cells (**b**). iP-NBD detected partially co-localizing with HDEL-RFP in ER (red arrowheads) and in non-ER cellular structures (white arrowheads). **c.** Co-staining of root epidermal cells with iP-NBD (green) and FM4-64 (red) monitored 15 minutes after co-treatment. White arrowheads indicate co-localization of iP-NBD and FM4-64 in vesicles. **d**. Co-localization of iP-NBD (green) and RabA1e-mCherry (red) endosome/recycling endosome marker. Upper panel: co-localization of iP-NBD with RabA1e in vesicles (white arrowheads) before BFA treatment. Lower panel: accumulation of iP-NBD and RabA1e in the endosomal compartments (blue arrowheads) formed in root epidermal cells treated with 50 µM BFA for 1 h. **e.** Partial co-localization of iP-NBD (green) with a cis-GA marker SYP32-mCherry in root epidermal cells. White arrowheads indicate overlapping signals. **f, g**. Partial co-localization of iP-NBD (green) with an integral GA membrane protein Got1p-mCherry (**f**) and TGN/early endosome marker VTI12-mCherry (**g**) in root epidermal cells. White arrowheads indicate overlapping signals. **h, i**. Non-overlapping signals of iP-NBD (green) and late endosome marker RabF2b-mCherry (red, **h**) or vacuolar marker VAMP711-mCherry (red, **i**) in root epidermal cells. Purple arrowheads indicate RabF2-mCherry stained endosomes (**h**) and VAMP711-mCherry vacuolar compartments (**i**). Scale bars = 5 µm.

Supplementary Fig 4

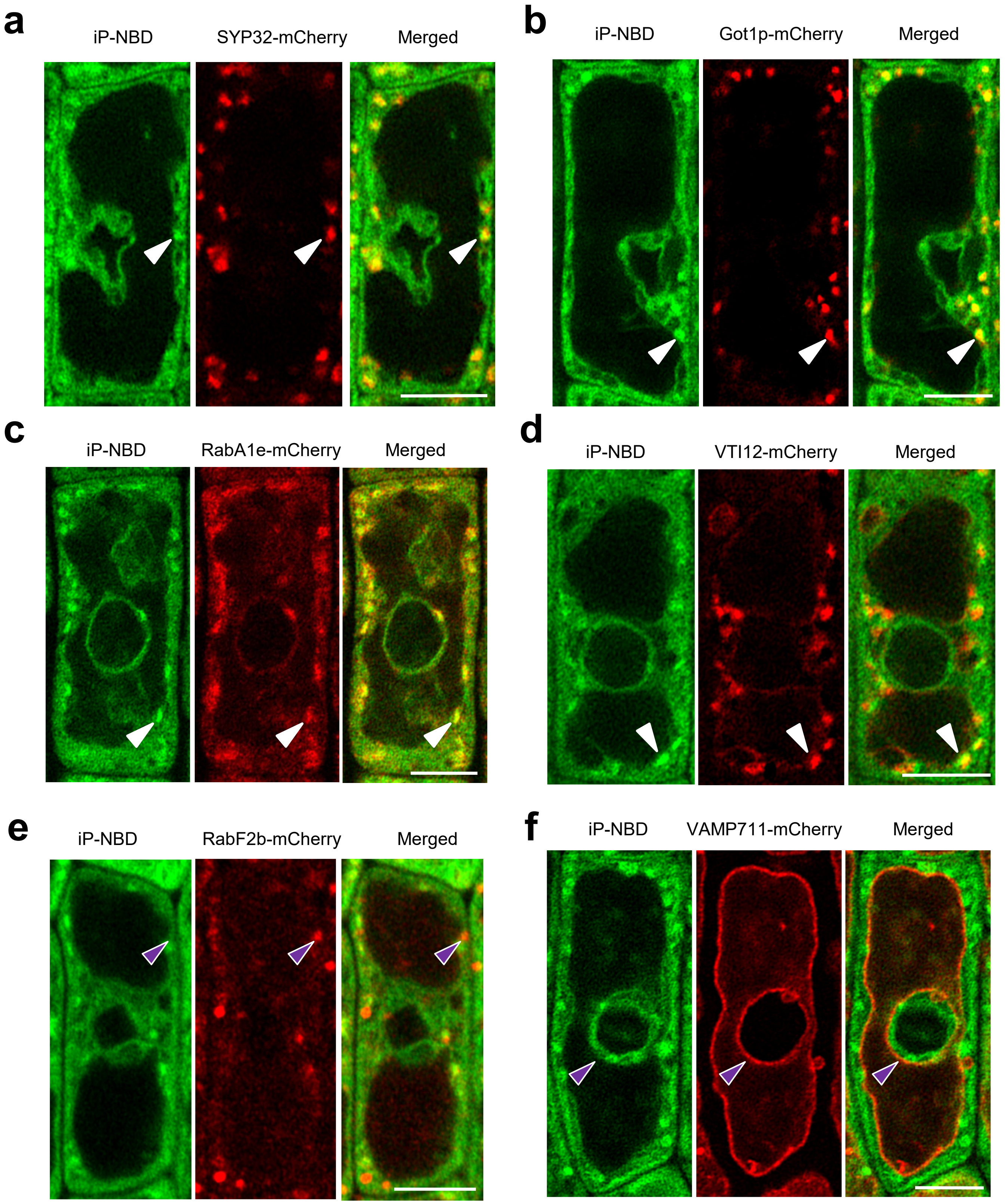

Supplementary Figure 4. Monitoring of iP-NBD subcellular localization in Arabidopsis lateral root cap cells.

a-d. Co-localization of iP-NBD (green) with a cis-GA marker SYP32-mCherry (red, **a**), an integral GA membrane protein Got1p-mCherry (red, **b**), endosome/recycling endosome marker RabA1e-mCherry (red, **c**) and TGN/early endosome marker VTI12-mCherry (**d**) in LRC cells. White arrowheads indicate overlapping signals. **e**, **f**. Non-overlapping signals of iP-NBD (green) and late endosome marker RabF2b-mCherry (**e**) or vacuolar marker VAMP711-mCherry (**f**, red) in LRC cells. Purple arrowheads indicate subcellular compartments visualised by specific markers. Scale bars = 5 μm.

Supplementary Fig 5

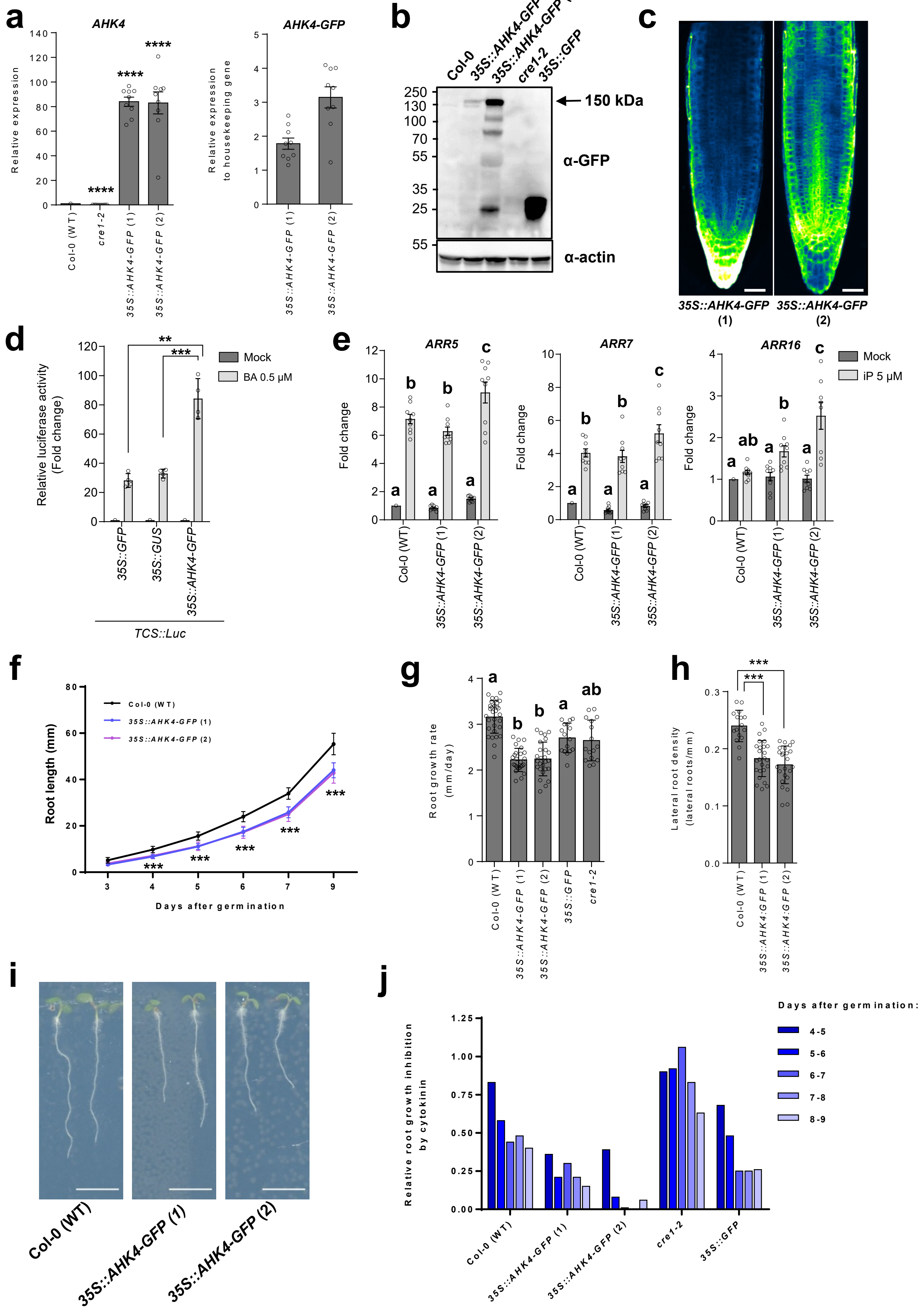

Supplementary Figure 5. **Analysis of CRE1/AHK4-GFP functionality *in vivo***.

a. Expression analysis of *AHK4* and *AHK4-GFP* in 5-day-old seedlings using quantitative RT-PCR. Relative expression of cytokinin reporter in two *35S::AHK4-GFP* independent lines was evaluated when compared to Col-0 (WT) (left panel), or housekeeping gene (right panel). Mean ± s.d., $p < 0.05$ by two-way ANOVA. $n = 9$ (3 technical replicates from 3 biological replicates) per condition. Specific primer pairs for CRE1/AHK4 (left graph) and CRE1/AHK4-GFP (right graph) were used. **b.** Western blot analysis of AHK4-GFP in total protein extracts from the two independent *35S::AHK4-GFP* lines. Col-0, *cre1-2, 35S::GFP* used as controls. Membranes were incubated with anti-GFP and anti-actin antibodies. Arrow marks expected molecular weight of AHK4-GFP (150 kDa). **c.** Monitoring of CRE1/AHK4-GFP signal in the meristematic zone of Arabidopsis root in two independent *35S::AHK4-GFP* lines. **d**. *TCS::LUCIFERASE* cytokinin reporter activity in Arabidopsis protoplasts co-transformed with CRE1/AHK4-GFP reporter is significantly upregulated in response to cytokinin (0.5 μM BA) when compared to protoplasts co-transformed with either *GFP* or *GUS* reporter only (mean ± s.d; *** $p < 0.001$ Student's t-test indicates significant difference when compared to protoplasts transformed with *35S::GUS,* $n = 4$). **e.** Expression of early cytokinin response genes *ARR5, ARR7* and *ARR16* in 5-day-old seedlings of Col-0 and the two independent *35S::AHK4-GFP* lines treated with 5µM iP or DMSO for 15 min. (mean ± s.d., $p < 0.05$ by two-way ANOVA. $n = 9$; 3 technical replicates from 3 biological replicates per condition). **f.** Root length of 3- to 9-day-old seedlings of control Col-0 (black) and two independent *35S::AHK4-GFP* lines (mean ± s.d., *** = $p < 0.001$ by Student's t-test, $n \geq 15$). **g.** Average root growth rate (mm/day) of control Col-0, two independent *35S::AHK4-GFP* lines, *35S::GFP* and *cre1-2* seedlings during 5 days (mean ± s.d.; $p < 0.01$ by ANOVA test, $n \geq 15$). **h**. Lateral root density (number of lateral roots/root length) was evaluated in 9-day-old seedlings of control Col-0 and two independent *35S::AHK4-GFP* lines (mean ± s.d.; *** = $p < 0.001$ by Student's t-test, $n \geq 15$). **i.** Representative images of 5-day-old seedlings of control Col-0 and two independent *35S::AHK4-GFP* lines. **j.** Relative inhibition of root growth by cytokinin in control Col-0, two independent *35S::AHK4-GFP* lines, *35S::GFP* and *cre1-2* seedlings. Root growth on medium with and without cytokinin (BA 1 µM) monitored during 5 days (day 4 to 9 after germination) and relative root growth inhibition per day calculated ($n = 10\text{-}15$ roots). Scale bars = 5 mm (**i**) and 10 µm (**c**).

Supplementary Fig 6

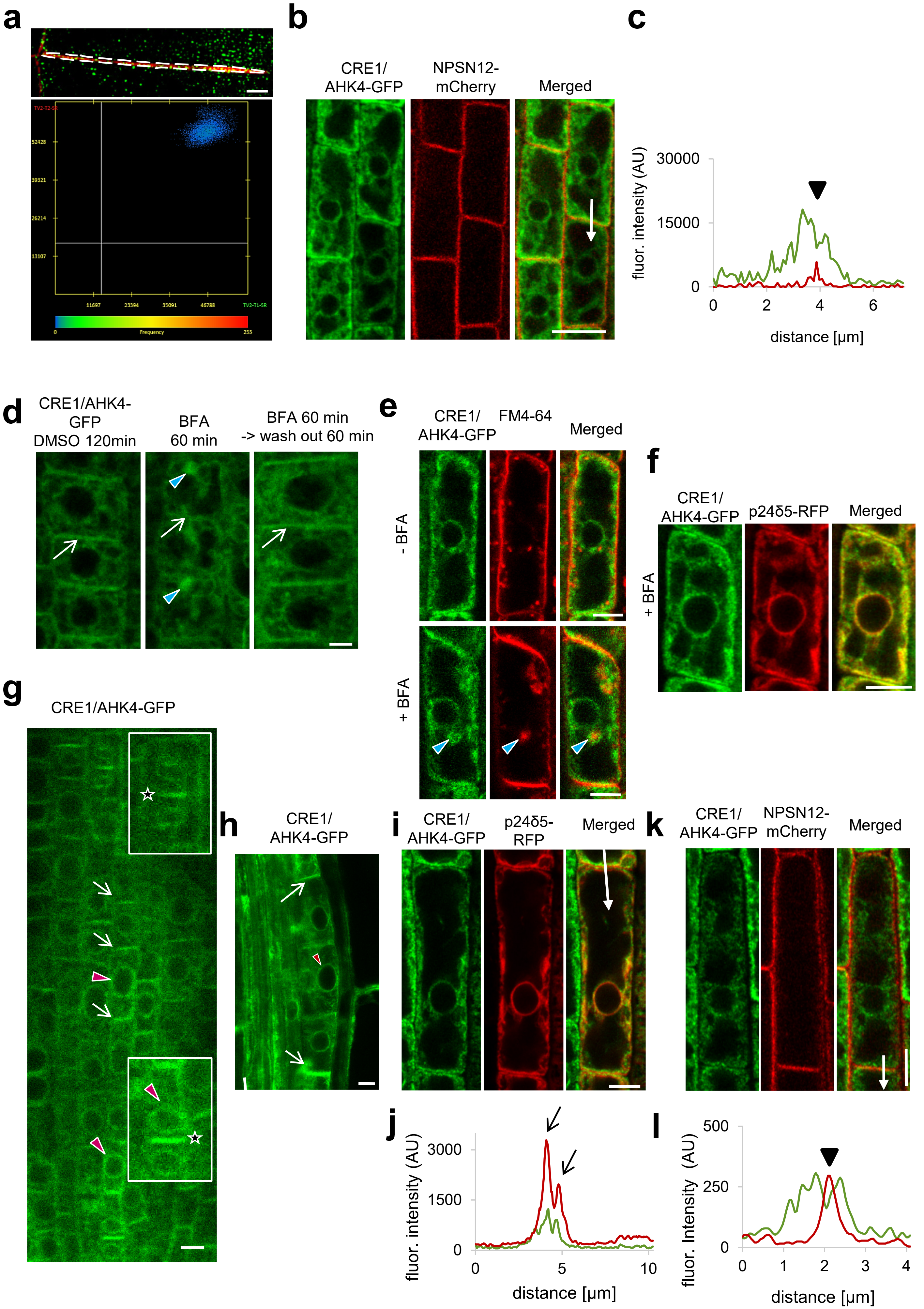

Supplementary Figure 6. CRE1/AHK4-GFP localization in Arabidopsis root cells.

a. Co-localization analysis of CRE1/AHK4 with the FM4-64 labelled plasma membrane in epidermal cells of the root meristem. Dotted line in upper image corresponded to the plasma membrane co-localization area. Bottom image shows scatter plot of marked the plasma membrane area in upper image. **b, c**. Monitoring of CRE1/AHK4-GFP cytokinin receptor (green) and the NPSN12-RFP plasma membrane reporter (red) in LRC cells (**b**). Profiles of fluorescence intensity of the plasma membrane marker (red line) and CRE1/AHK4-GFP (green line) were measured along the white line (**b**) starting from upper end (0 µm) towards the arrowhead. Peak of NPSN12-mCherry fluorescence maxima at the plasma membrane does not overlap with CRE1/AHK4-GFP fluorescence maximum (black arrowhead, **c**). **d.** Re-location of CRE1/AHK4-GFP cytokinin receptor from the endosomal compartments to the plasma membrane after BFA wash-out in the root epidermal cells. Blue arrowheads indicate CRE1/AHK4 in the BFA-bodies. Note the attenuated AHK4-GFP signal at the plasma membrane and its re-localization back after 60 min wash-out of BFA (white arrows). **e, f.** Monitoring of CRE1/AHK4-GFP (green), FM4-64 (red) (**e**) and p24δ5-RFP (red) (**f**) in LRC cells treated for 1 h with 50 µM BFA. FM4-64, but not p24δ5-RFP detected in the BFA endosomal compartments (blue arrowheads). In some cells of LRC CRE1/AHK4-GFP signal scattered around BFA bodies detected (**e**). **g, h.** Monitoring of the CRE1/AHK4-GFP signal in cells of provasculature at the root meristematic zone (**g**) and developing lateral root primordia (**h**). Black stars mark AHK4-GFP signal at cell plate of diving cells, white arrows and red arrowheads point to the plasma membrane and ER structures, respectively. **i-l**. Monitoring of CRE1/AHK4-GFP cytokinin receptor (green), ER-marker p24δ5-RFP (red) (**i**, **j**) and the plasma membrane marker NPSN12-mCherry (red) (**k**, **l**) in differentiated epidermal cells at the root elongation zone. Profile of fluorescence intensity of p24δ5-RFP ER marker (red line) and CRE1/AHK4-GFP (green line) (**j**) was measured along the white line (**i**) starting from upper end (0 µm) towards the arrowhead. Peaks of p24δ5-RFP fluorescence maxima correlate with endoplasmic reticulum signal and overlap with CRE1/AHK4-GFP signal maxima (black arrows; **j**). Profile of fluorescence intensity of the NPSN12-mCherry plasma membrane marker (red line) and CRE1/AHK4-GFP (green line) (**l**) was measured along the white line (**k**) starting from upper end (0 µm) towards the arrowhead. Peak of NPSN12-mCherry fluorescence maxima at the plasma membrane signal does not overlap with CRE1/AHK4-GFP signal maxima in the epidermal cells (black arrowhead; **l**). Scale bars = 5 µm (**b, d-i, k**) and 2 µm (**a**).

Supplementary Fig 7

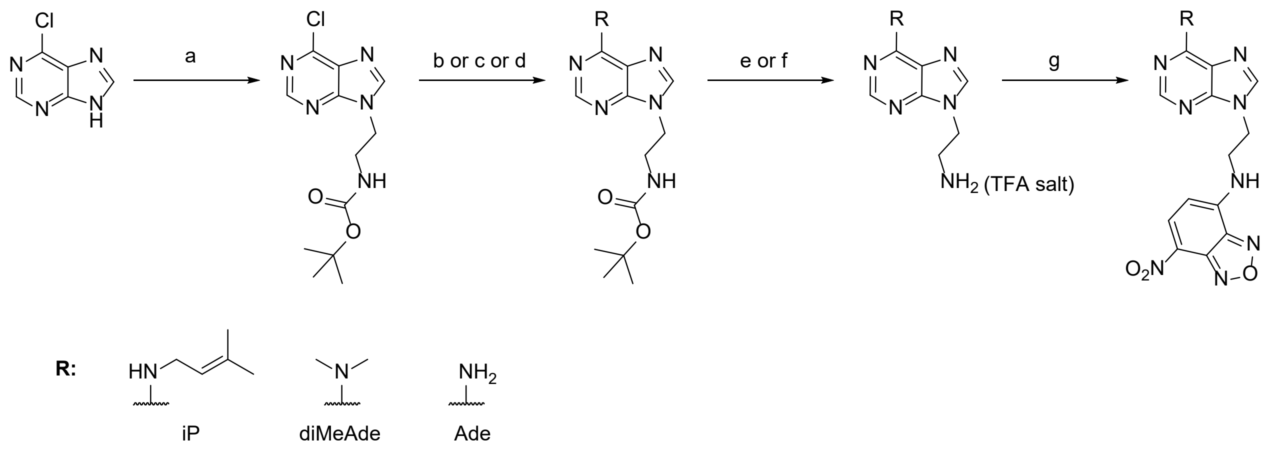

Supplementary Figure 7. Reaction scheme for preparation of fluorescently labelled probes for confocal microscopy studies. a) *N*-Boc-ethanolamine, PPh_3, DIAD, THF, rt, 2 h, (68%); b) 3-methylbut-2-enylamine.HCl, Et_3N, *n*PrOH, reflux, 4 h, (93%); c) Me_2NH.HCl, Et_3N, *n*PrOH, reflux, 4 h, (97%); d) ammonium hydroxide, EtOH, 95 °C, overnight, (76%); e) Dowex 50W X8, DCM, reflux followed by 4 M methanolic ammonia, rt, overnight, (iP - 90%, diMeAde - 87%); f) TFA, DCM, rt, overnight, (Ade.TFA salt - 95%); g) 4-chloro-7-nitro-1,2,3-benzoxadiazole, $NaHCO_3$, MeOH, 50 °C, 1 h followed by rt overnight (iP - 52%, diMeAde - 75%, Ade - 68%).

Supplementary Table 1

iP-NBD colocalization with marker lines			Pearson's correlation coefficient	
line	compartment	fusion protein	LRC cells	Meristematic epidermal cells
p24δ5-RFP	*ER*	p24δ5-RFP	0.61 ± 0.02	0.50 ± 0.02
W18	GA	Got1p-mCherry	0.67 ± 0.02	0.56 ± 0.03
W22	GA	SYP32-mCherry	0.69 ± 0.02	0.53 ± 0.03
W34	*endosomes, RE*	RabA1e-mCherry	0.39 ± 0.06	0.43 ± 0.04
W13	*TGNIEE*	VTI12-mCherry	0.28 ± 0.04	0.32 ± 0.04
W2	*LE/PVC*	RabF2b-mCherry	-0.15 ± 0.03	-0.18 ± 0.05
W9	*vacuoles*	VAMP711-mCherry	-0.12 ± 0.04	0.00 ± 0.00

Supplementary Table 1. Pearson's correlation coefficients of iP-NBD co-localization with marker lines. Quantification of co-localization of iP-NBD staining with various markers for different subcellular compartments (ER – endoplasmic reticulum, GA – Golgi apparatus, RE – recycling endosomes, TGN/EE – trans-Golgi network, early endosomes, LE/PVC – late endosomes/pre-vacuolar compartments). Average ± s.e., n = 8-25.

Supplementary Methods

Receptor activation assay. The receptor activation assays were conducted using the *E. coli* strain KMI001 harbouring either the plasmid *pIN-III-AHK4* or *pSTV28-AHK3,* which express the Arabidopsis histidine kinases CRE1/AHK4 or AHK3[2,3]. Bacterial strains were kindly provided by Dr. T. Mizuno (Nagoya, Japan). The assays were performed in liquid M9 media enriched with 0.1% casamino acids (casein hydrolysate, acid hydrolyzed) (Merck, Germany) and antibiotics (ampicilin/carbenicilin, and chloramphenicol for culturing of CRE1/AHK4- and AHK3-expressing bacteria, respectively) using 96-well plate format[4]. First, the *E. coli* strains were grown overnight in M9 medium (containing 0.1% casamino acids and antibiotics) to OD600 ~ 1. These precultures were diluted 1:600 in M9 medium (containing 0.1% casamino acids and antibiotics) and a 96-well plate was filled up with 200 µL of the diluted culture per well. 1 µL stock solution of either the tested compound or solvent control was added to reach desired final concentrations and the cultures were further grown while shaking for 17 h at 25°C. 50 µL aliquots of the culture were transferred to wells of a 96-well plate containing 2 µL of 25 mM 4-methyl umbelliferyl galactoside (MUG, Sigma)/well. The plate was subsequently incubated for 30 min at 37°C and the reaction was stopped by adding 100 µL glycine carbonate stop buffer (133 mM glycine, 83 mM Na_2CO_3, pH 10.7). OD600 of remaining culture was determined using a spectrophotometer. Fluorescence was measured using Synergy H4 Multi-Mode Microplate Reader (BioTek, USA) at the excitation and emission wavelengths of 365 and 460 nm, respectively. β-galactosidase activity was calculated as nmol 4-methylumbelliferone $OD600^{-1}$ h^{-1}.

***ARR5::GUS* reporter gene assay.** Arabidopsis *ARR5::GUS* transgenic seeds were surface-sterilized and plated on 0.5x MS medium with 0.1% (w/v) sucrose and 0.05% (w/v) MES–KOH (pH 5.7) in 24-well plates, 20 seeds per well. The plates were stratified for 3 days at 4 °C in darkness and then grown under long-day conditions (16 h light/8 h dark) at 22 °C in a growth chamber. To the wells containing 3-day-old seedlings, BA and/or tested compound or DMSO (solvent control, final concentration 0.1%) was added and the seedlings were grown for an additional 16 h. After that the seedlings were flash frozen in liquid nitrogen. Quantitative determination of GUS activity in seedlings extracts was performed 4-methylumbelliferyl glucuronide as a substrate[38] and the fluorescence was measured using Synergy H4 Multi-Mode Microplate Reader (BioTek, USA) at excitation and emission wavelengths of 365 and 450 nm,

respectively. The GUS activity was expressed in relative fluorescence units (RFU) of MU per seedling.

Western blot analysis of AHK4-GFP protein levels. To evaluate levels of the CRE1/AHK4-GFP protein expression, total protein extract was obtained from 5-day-old seedlings extracted with 1x Laemmli buffer (2-Mercaptoethanol, 0.1% Bromophenol blue, 0.0005% Glycerol, 10% SDS (electrophoresis-grade), 2% Tris-HCl, 63 mM pH 6.8). Protein extracts were used for SDS–PAGE using gels acrylamide 10%, and blotted to PVDF transfer membrane (Millipore). Two hours incubation with monoclonal mouse anti-GFP (JL-8, Clontech) 1:5000; or 1 hour incubation with monoclonal mouse anti-Actin (10-B3, Sigma) 1:5000 for protein loading control; as a primary antibodies were used. As a secondary antibody, 1 hour incubation sheep anti-mouse IgG Horseradish Peroxidase - Linked F(ab′)2 Fragment (NA9310, GE Healthcare) 1:15000 was used. SuperSignal™ West Femto Maximum Sensitivity Substrate Thermo Fisher (4 minutes incubation) was used for detection of the signal.

TCS reporter expression in planta. 5-day-old seedlings expressing cytokinin signaling reporter *TCSn::ntdT:tNOS* (line *TCSn::ntdT:tNOS-pDR5v2::n3GFP*) were transferred on non- or with cytokinin (1 μM iP, 1μM iP-NBD or iP 1 μM + 1 μM iP-NBD) supplemented Murashige and Skoog media for 6 or 15 h. The TCS:ntdT expression (red, LUT inferno) in root tip was monitored using confocal microscope. Fluorescence intensity (arbitrary numbers) of TCS:ntdT signal was measured in cells of provasculature at the root tip meristematic zone (n = 10 roots per treatment).

AtCKX2 activity measurement. The recombinant enzyme AtCKX2 was obtained from cell free medium of *P. pastoris* pGAPZα::AtCKX2 strain[5]. The yeasts were precultivated in YPD medium (1 % yeast extract, 2 % pepton) complemented with 2 % glucose and 100 μg mL^{-1} zeocin, at 30 °C for 72 h in dark, while shaking. Afterthat the preculture was diluted 25-times into fresh YPD medium complemented with 2 % glucose and cultivated at the same conditions for another 24 h. Cells were removed by centrifugation at 10.000 rpm and 4 °C for 10 min. pH of the cell free medium containing enzyme was adjusted to pH 8 using 2 M Tris/HCl, pH 8 and then sterilized by passing through 0.2 μm filter. The enzymatic activity determination was done kinetically by the 2,6-dichlorophenolindophenol (DCPIP) method[6] adopted for screening in microtitre plates. Each well was filled with 100 μL of reaction mixture containing 100 mM KH_2PO_4 buffer, pH 7.4, 0.2 mM DCPIP (to reach starting absorbance 1.0 at 590 nM), 2 mM

EDTA and 0.2 mg mL^{-1} BSA. iP or iP-NBD were added from 10 mM stock solutions in DMSO to reach the desired final concentration of 1.6 – 200 mM. The same volume of DMSO was used as a blank. Reaction was started by addition of 100 μL enzyme diluted from the source in 0.1 M Tris/HCl, pH 8 to reach activity 50 pkat mL^{-1}. Absorption at 590 nm was recorded in 40 s intervals for 10 min at 37 °C in Synergy H4 Hybrid Multi-Mode Microplate reader (BioTek, USA). From Average reaction rates (mOD/min) were determined from linear sections and the reaction rate of the blank was subtracted. The enzymatic kinetic parameters V_{max} and K_m were calculated using GraphPad Prism 5.1.

***In vitro* pH stability measurement.** The pH stability of iP-NBD was analyzed by HPLC-PDA Waters Alliance 2695 Separations Module (Waters, Manchester, UK); analytes were detected at scanning range 210-700 nm using Waters 2996 PDA detector (Waters, Manchester, UK). Solution of iP-NBD (1 mM; DMSO) was prepared and diluted to 10 μM in the McIlvaine buffer (ref) with appropriate pH (4, 5, 6, 7 or 8). Diluted sample was either immediately injected (0 h) onto a reversed phase column (Symmetry C18, 5 μm, 150 mm × 2.1 mm; Waters, Milford, MA, USA), or left incubated at 21 °C for 0.5 h, 6 h or 16 h and then injected on the column. At flow-rate of 0.3 mL min^{-1}, the following binary gradient was used: 0 min, 90 % A; 0–14 min, linear gradient to 10 % A; 14–20 min, isocratic elution of 10 % A; 20–24 min, linear gradient to 90 % A; 24-26 min, isocratic elution of 90 % A, where A was 15 mM formic acid adjusted to pH 4 with ammonium and B was 100 % methanol. Chromatograms were generated under the 270 nm wavelength. Relative peak areas of iP-NBD corresponding to samples incubated for selected time periods in the McIlvaine buffer were calculated as percentages of the corresponding peak areas obtained for the control sample with the same dilution (0h); all peak areas were measured at least in triplicates.

LC-MS/MS method for detection of iP-NBD biological stability. To study iP-NBD stability *in vivo* 5 µM iP-NBD was applied to *Arabidopsis* (*Ler*) cell suspension and in the timeframe of 0.5 - 5 h samples were taken and deep-frozen for subsequent quantitative LC-MS/MS analysis of iP-NBD and Ade-NBD (the expected product of side-chain cleavage by endogenous CKXs) contents. The samples (around 50 mg fresh weight) were extracted in 1.0 mL of modified Bieleski buffer (60% MeOH, 10% HCOOH and 30% H_2O[7]) together with 0.01 pmol of N9-NBD-labelled dimethyladenine (diMeAde-NBD) added as internal standard to validate LC-MS/MS determination. The extracts were purified using the Oasis MCX column (30 mg/1 mL, Waters) and targeted analytes were eluted using 0.35 M NH_4OH in 60% (v/v) MeOH solution[8].

The purified samples were eluted using a reversed-phase column (Acquity UPLC® BEH C18, 1.7 µm, 2.1 × 150 mm, Waters) with a 26 min gradient comprised of methanol (A) and 15 mM ammonium formate pH 4.0 (B) at a flow rate of 0.25 mL/min and column temperature of 40 °C[9]. The binary linear gradient of 0-7 min 5:95 A:B, 16 min 20:80 A:B, 23 min 50:50 and 26 min 100:0 was used, after which the column was washed with 100% methanol for 1 min and re-equilibrated to initial conditions for 3 min. The effluent was introduced into the MS system with the following optimal settings: source/desolvation temperature 150/600 °C, cone/desolvation gas flow 150/1000 l h^{-1}, capillary/cone voltage 3500/25 V, collision energy 20 eV and collision gas flow (argon) 0.15 mL min^{-1}. Quantification and confirmation of the NBD-labelled compounds were obtained by the multiple reaction monitoring mode using the following mass transitions: 410>342, 342>136 and 370>164 for iP-NBD, Ade-NBD and diMeAde-NBD, respectively. All chromatograms were analyzed with MassLynx software (version 4.1; Waters Corporation) and the compounds were quantified according to the internal standard added.

Estimation of iP-NBD fluorescence properties. Fluorescence spectrum reaching the maximal fluorescence intensities in 456 nm (ex) and 528 nm (em) was obtained with 100 µM iP-NBD dissolved in 100% ethanol by relative fluorescence intensity scanning in the range of 300-700 nm using Synergy H4 Multi-Mode Microplate Reader (BioTek, USA).

Luciferase transient expression assay. The luciferase transient expression assays were performed on protoplasts isolated from 4-days-old Arabidopsis root suspension culture. Protoplasts were isolated in enzyme solution (1% cellulose; Serva, 0.2% Macerozyme; Yakult in B5-0.34M glucose-mannitol solution; 2.2 g MS with vitamins, 15.25 g glucose, 15.25 g mannitol, H_2O to 500 ml pH to 5.5 with KOH) with slight shaking for 3-4 h and centrifuged at 800g for 5 min. The pellet was washed with B5-0.34M glucose-mannitol solution and resuspended in B5-0.34M glucose-mannitol solution to a final concentration of $2x10^5$ protoplasts per 50 µl. Protoplasts were co-transfected with 3 µg of a reporter plasmid expressing *Firefly* luciferase (*f*LUC), 2 µg of normalization plasmid containing the *Renilla* luciferase (*r*LUC) and 10 µg of p2GW7,0 plasmid carrying either cytokinin receptor (*35S::AHK4-GFP*) or reporter only (*35S::GUS* or *35S::GFP*) constructs. DNAs were gently mixed together with 50 µl of protoplast suspension and 60 µl of PEG solution (0.1M $Ca(NO_3)_2$, 0.45M Mannitol, 25% PEG 6000) and incubated in the dark for 30min. Then 140 µl of 0.275M $Ca(NO_3)_2$ solution was added to wash off PEG, wait for sedimentation of protoplasts and remove 240 µl

of supernatant. The protoplast pellet was resuspended in 200 µl of B5-0.34M glucose-mannitol solution and incubated for 16 h with either 0.5 µM BA or in mock solution in the dark at room temperature. After transfection, protoplasts were centrifuged at 1200g for 5 min and lyzed; *f*LUC and *r*LUC activities were determined with the Dual-Luciferase reporter assay system (Promega). Variations in transfection efficiency and technical errors were corrected by normalization of *f*LUC by the *r*LUC activities. The mean value was calculated from four measurements and experiment was repeated three times.

Root growth analysis. Root growth and lateral root density were measured on seedlings grown vertically on Murashige and Skoog medium. Images were taken with a vertically positioned scanner, EPSON perfection v800 Photo. Root growth rate was evaluated using 5-day-old seedlings ($n \geq 16$). Lateral root density quantification was performed using 9-day-old seedlings ($n \geq 16$). Relative root growth inhibition by cytokinin was measured per day (24h) and was calculated as a ratio of 1 µM BA to DMSO treated Arabidopsis seedlings in the corresponding days after germination. The statistical significance was evaluated with the Student's t-test and two-way ANOVA.

Synthesis of fluorescently-labelled compounds. The fluorescently labelled compounds used in the studies were prepared according to the reaction scheme (Fig. S7). Two carbon linker terminated with amino group suitable for consecutive NBD fluorophore attachment was coupled to the purine *N*9 position by the reaction of 6-chloropurine with *N*-Boc-ethanolamine under Mitsunobu conditions[1]. Later, nucleophilic substitution of C6 chlorine with appropriate amines in boiling alcohols followed by Boc protective group cleavage provided purine intermediates for fluorescent labelling. 4-chloro-7-nitro-1,2,3-benzoxadiazol was used as NBD donor and was linked to the purine primary amino group in MeOH under basic conditions. Physico-chemical characterization of the synthesized compounds was done according to[1]. Thin-layer chromatography was carried out on Silica 60 F_{254} plates (Merck) using *n*-PrOH/ammonium hydroxide/water (55:10:35, v/v) or $CHCl_3$/MeOH (9:1, v/v) as a developing systems and the spots were detected by UV light (254 nm) and/or by ninhydrin staining (1.5% (w/v) ninhydrin in *n*-butanol containing 3 % of AcOH). The column chromatography purification was performed on silica Davisil 40-63 micron (Grace Davision). The chromatographic purity and mass of prepared compounds was determined using an Alliance 2695 separation module (Waters) linked simultaneously to a DAD detector PDA 996 (Waters) and a Q-Tof micro (Waters) benchtop quadrupole orthogonal acceleration time-of-flight

tandem mass spectrometer. Samples were dissolved in DMSO and diluted to a concentration of 10 μg.mL^{-1} in initial mobile phase. The samples (10 μL) were injected on a RP-column Symmetry C18 (150 mm × 2.1 mm × 3.5 μm, Waters) and separated at a flow rate of 0.2 mL.min^{-1} with following binary gradient: 0 min, 10% B; 0-24 min, a linear gradient to 90% B, followed by 10 min isocratic elution of 90% B. 15 mM formic acid adjusted to pH 4.0 by ammonium hydroxide was used as solvent (A) and methanol as the organic modifier - solvent (B). The eluent was introduced into the DAD (scanning range 210-400 nm, with 1.2 nm resolution) and an ESI source (source temperature 110 °C, capillary voltage +3.0 kV, cone voltage +20 V, desolvation temperature 250 °C). Nitrogen was used both as desolvation gas (500 L.h^{-1}) as well as cone gas (50 L.h^{-1}). The data was obtained in positive ionization mode and were acquired in the 50-1000 m/z range. ^{1}H and ^{13}C NMR spectra were recorded on Jeol ECA-500 operating at a frequency of 500 MHz (^{1}H) and 125 MHz (^{13}C), respectively. Samples were prepared by dissolving substances in DMSO-d_6 and the chemical shifts were calibrated to residual solvent peak (2.49 ppm for proton) and DMSO-d_6 (39.5 ppm for carbon). For assignment of ^{1}H and ^{13}C NMR signals 2D NMR spectra such as HMQC, HMBC, and COSY were also measured.

tert-butyl [2-(6-amino-9_H_-purin-9-yl)ethyl]carbamate

tert-butyl [2-(6-amino-9*H*-purin-9-yl)ethyl]carbamate (0.5 g, 1.68 mmol) was heated in closed vessel with ammonium hydroxide (5 mL) and EtOH (5 mL) at 95 °C overnight. After evaporation of solvents the residue was purified by silica column chromatography using $CHCl_3$/MeOH (9:1, v/v) as a mobile phase. White solid, yield 76%. HPLC purity 99.9, ESI$^+$-MS 279 (100, [M+H]$^+$), ^{1}H-NMR (500 MHz, DMSO-d_6) δ (ppm): 1.30 (s, 9H, Boc $(C\underline{H}_3)_3$), 3.32 (q, J = 5.9 Hz, 2H, $CH_2C\underline{H}_2NHBoc$), 4.16 (t, J = 6.0 Hz, 2H, $C\underline{H}_2CH_2NHBoc$), 6.96 (t, J = 5.7 Hz, 1H, $CH_2CH_2N\underline{H}Boc$), 7.15 (s, 2H, Ade NH_2), 7.99 (s, 1H, pur H8), 8.11 (s, 1H, pur H2). ^{13}C-NMR (125 MHz, DMSO-d_6) δ (ppm): 28.1 (Boc $(\underline{C}H_3)_3C$), 39.2 (HMQC based, $CH_2\underline{C}H_2NHBoc$), 42.7 ($\underline{C}H_2CH_2NHBoc$), 77.8 (Boc $(CH_3)_3\underline{C}$), 118.7 (pur C5), 140.8 (pur C8), 149.6 (pur C4), 152.2 (pur C2), 155.5 (Boc $\underline{C}O$), 155.9 (pur C6).

9-(2-aminoethyl)adenine trifluoroacetate

tert-butyl [2-(6-amino-9*H*-purin-9-yl)ethyl]carbamate (0.35 g, 1.26 mmol) was added to a mixture of DCM (10 mL) and TFA (0.5 mL, 6.54 mmol) and stirred at room temperature overnight. Reaction mixture was evaporated under reduced pressure and the residue was treated with Et_2O to obtain white solid. Yield 95%, HPLC purity 99.9, ESI$^+$-MS 179 (100, [M+H]$^+$),

^{1}H-NMR (500 MHz, DMSO-d_6) δ (ppm): 3.36 (s, 2H, $CH_2C\underline{H}_2NH_2$), 4.47 (t, J = 5.7 Hz, 2H, $C\underline{H}_2CH_2NH_2$), 8.13 (s, 3H, $CH_2CH_2N\underline{H}_3^+$), 8.34 (s, 1H, pur H8), 8.43 (s, 1H, pur H2), 8.72 (s, 2H, Ade NH_2). ^{13}C-NMR (125 MHz, DMSO-d_6) δ (ppm): 38.3 ($CH_2\underline{C}H_2NH_2$), 41.4 ($\underline{C}H_2CH_2NH_2$), 116.5 (q, $^{1}J_F$ = 296.3 Hz, $\underline{C}F_3COOH$) 118.5 (pur C5), 142.9 (pur C8), 147.5 (pur C2), 149.3 (pur C4), 152.3 (pur C6), 158.8 (q, $^{2}J_F$ = 33.5 Hz, $CF_3\underline{C}OOH$).

Ade-NBD

A suspension of 9-(2-aminoethyl)adenine trifluoroacetate (0.1 g, 0.34 mmol) and $NaHCO_3$ (0.101 g, 1.20 mmol) was stirred in MeOH (3 mL) for 1 h. Then, NBD-chloride (0.084 g, 0.41 mmol) was added and the reaction mixture was heated in the dark at 65 °C for one hour and then stirred at room temperature overnight. Resulting solid was filtered, washed with ice cold MeOH (5 × 1 mL) and water (3 × 1 mL). The product was purified by silica column chromatography using $CHCl_3$/MeOH as a mobile phase with MeOH gradient.

Redish-brown solid, yield 68 %, HPLC purity 99.9, ESI$^+$-MS 342 (100, $[M+H]^+$), ^{1}H-NMR (500 MHz, DMSO-d_6) δ (ppm): 3.92 (s, 2H, $CH_2C\underline{H}_2NHNBD$), 4.46 (s, 2H, $C\underline{H}_2CH_2NHNBD$), 6.44 (d, J = 8.3 Hz, 1H, NBD H6), 7.19 (s, 2H, NH_2), 8.04 (s, 1H, pur C2), 8.09 (s, 1H, pur H8), 8.46 (d, J = 8.9 Hz, 1H, NBD H5), 9.48 (s, 1H, $CH_2CH_2N\underline{H}NBD$). ^{13}C-NMR (125 MHz, DMSO-d_6) δ (ppm): 41.3 ($\underline{C}H_2CH_2NHNBD$), 42.8 ($CH_2\underline{C}H_2NHNBD$), 99.3 (NBD C6), 118.7 (pur C5), 121.3 (NBD), 137.7 (NBD C5), 141.0 (pur C8), 144.0 (NBD), 144.4 (NBD), 144.9 (NBD), 149.7 (pur C4), 152.3 (pur C2), 155.9 (pur C6).

Supplementary references

1 Kubiasová, K. et al. Design, synthesis and perception of fluorescently labeled isoprenoid cytokinins. *Phytochemistry* **150**, 1-11 (2018).
2 Suzuki, T. et al. The Arabidopsis sensor His-kinase, AHK4, can respond to cytokinins. *Plant Cell Physiol.* **42**, 107-113 (2001).
3 Yamada, H. et al. The Arabidopsis AHK4 histidine kinase is a cytokinin-binding receptor that transduces cytokinin signals across the membrane. *Plant & Cell Physiol.* **42**, 1017-1023 (2001).
4 Spíchal, L. Bacterial assay to study plant sensor histidine kinases. *Methods Mol. Biol.* **779**, 139-147 (2011).
5 Kowalska, M. et al. Vacuolar and cytosolic cytokinin dehydrogenases of Arabidopsis thaliana: Heterologous expression, purification and properties. Phytochemistry **71**, 1970–1978 (2010).
6 Bilyeu, K. D. et al. Molecular and biochemical characterization of a cytokinin oxidase from maize. *Plant Physiol.* **125**, 378-386 (2001).
7 Hoyerová, K. et al. Efficiency of different methods of extraction and purification of cytokinins. *Phytochemistry* **67**, 1151-1159 (2006).

8 Dobrev, P. I. & Kamínek, M. Fast and efficient separation of cytokinins from auxin and abscisic acid and their purification using mixed-mode solid-phase extraction. *J. Chromatogr. A.* **950**, 21-29 (2002).

9 Svačinová, J. et al. A new approach for cytokinin isolation from Arabidopsis tissues using miniaturized purification: pipette tip solid-phase extraction. *Plant Methods* **8**, 17, (2012).
