# Supplementary material for: Cytokinin fluoroprobe reveals multiple sites of cytokinin perception at plasma membrane and endoplasmic reticulum
Source: Nat Commun. 2020 Aug 27;11:4285. doi: 10.1038/s41467-020-17949-0 (PMC7452891; doi:10.1038/s41467-020-17949-0)
Supplement: Supplementary file 3 — Descriptions of Additional Supplementary Files [file 41467_2020_17949_MOESM3_ESM.docx]

**Descriptions of Additional Supplementary Files**

**Supplementary Movie 1**

**Description:**  Real time monitoring of the CRE1/AHK4-GFP localization during lateral root primordia (LRP) development. LRP at stage II was monitored for 6 h. CRE1/AHK4-GFP at the cell plate of dividing cells is indicated by white arrows.
