# Supplementary material for: Cytokinin fluoroprobe reveals multiple sites of cytokinin perception at plasma membrane and endoplasmic reticulum
Source: Nat Commun. 2020 Aug 27;11:4285. doi: 10.1038/s41467-020-17949-0 (PMC7452891; doi:10.1038/s41467-020-17949-0)
Supplement: Supplementary file 5 — Reporting Summary [file 41467_2020_17949_MOESM5_ESM.pdf]

nature research

Corresponding author(s): Eva Benková, Lukáš Spíchal, Ondřej Plíhal

Last updated by author(s): Jul 6, 2020

Reporting Summary

Nature Research wishes to improve the reproducibility of the work that we publish. This form provides structure for consistency and transparency in reporting. For further information on Nature Research policies, see Authors & Referees and the Editorial Policy Checklist .

Statistics

For all statistical analyses, confirm that the following items are present in the figure legend, table legend, main text, or Methods section.

n/a	Confirmed	
☐	☒	The exact sample size (n) for each experimental group/condition, given as a discrete number and unit of measurement
☐	☒	A statement on whether measurements were taken from distinct samples or whether the same sample was measured repeatedly
☐	☒	The statistical test(s) used AND whether they are one- or two-sided<br>*Only common tests should be described solely by name; describe more complex techniques in the Methods section.*
☒	☐	A description of all covariates tested
☒	☐	A description of any assumptions or corrections, such as tests of normality and adjustment for multiple comparisons
☐	☒	A full description of the statistical parameters including central tendency (e.g. means) or other basic estimates (e.g. regression coefficient) AND variation (e.g. standard deviation) or associated estimates of uncertainty (e.g. confidence intervals)
☒	☐	For null hypothesis testing, the test statistic (e.g. F, t, r) with confidence intervals, effect sizes, degrees of freedom and P value noted<br>*Give P values as exact values whenever suitable.*
☒	☐	For Bayesian analysis, information on the choice of priors and Markov chain Monte Carlo settings
☒	☐	For hierarchical and complex designs, identification of the appropriate level for tests and full reporting of outcomes
☐	☒	Estimates of effect sizes (e.g. Cohen's d, Pearson's r), indicating how they were calculated

Our web collection on statistics for biologists contains articles on many of the points above.

Software and code

Policy information about availability of computer code

Data collection	Image data was collected using the Zeiss Zen (version 2011) software. For phenotyping, images were taken with EPSON perfection v800 Photo scanner.
Data analysis	Images were analysed using ImageJ (National Institute of Health, http://rsb.info.nih.gov/ij), Photoshop 6.0/CS and GraphPad Prism 8.

For manuscripts utilizing custom algorithms or software that are central to the research but not yet described in published literature, software must be made available to editors/reviewers. We strongly encourage code deposition in a community repository (e.g. GitHub). See the Nature Research guidelines for submitting code & software for further information.

Data

Policy information about availability of data

All manuscripts must include a data availability statement. This statement should provide the following information, where applicable:

- Accession codes, unique identifiers, or web links for publicly available datasets
- A list of figures that have associated raw data
- A description of any restrictions on data availability

All source data for the results in the manuscript are provided in the Material and Methods section.

Field-specific reporting

Please select the one below that is the best fit for your research. If you are not sure, read the appropriate sections before making your selection.

☒ Life sciences ☐ Behavioural & social sciences ☐ Ecological, evolutionary & environmental sciences

Life sciences study design

All studies must disclose on these points even when the disclosure is negative.

Sample size	Sample sizes were estimated as described in Material and Methods and in the Figure legends. At least 3 different images were analyzed per condition.
Data exclusions	No data sets were excluded from the analyses reported.
Replication	The results were replicated in 3 biological replicates, including at least 3 technically independent replications.
Randomization	Different treatments, mutants and conditions were analyzed in a randomized way.
Blinding	Blinding was not relevant for this study.

Reporting for specific materials, systems and methods

We require information from authors about some types of materials, experimental systems and methods used in many studies. Here, indicate whether each material, system or method listed is relevant to your study. If you are not sure if a list item applies to your research, read the appropriate section before selecting a response.

Materials & experimental systems

n/a	Involved in the study	
☐	☒	Antibodies
☒	☐	Eukaryotic cell lines
☒	☐	Palaeontology
☒	☐	Animals and other organisms
☒	☐	Human research participants
☒	☐	Clinical data

Methods

n/a	Involved in the study	
☒	☐	ChIP-seq
☒	☐	Flow cytometry
☒	☐	MRI-based neuroimaging

Antibodies

Antibodies used	monoclonal mouse anti-GFP (JL-8, Clontech) 1:5000; monoclonal mouse anti-Actin (10-B3, Sigma) 1:5000. As a secondary antibody, sheep anti-mouse IgG Horseradish Peroxidase - Linked F(ab')2 Fragment (NA9310, GE Healthcare) 1:15000 was used.
Validation	The specificity of antibodies used in this publication was verified in previous publications.
